# CAR T cells targeting the glycoprotein GD2 show potent antitumor efficacy in high-risk ependymoma models

**DOI:** 10.1172/JCI193332

**Published:** 2025-11-25

**Authors:** Antonio Carlos Tallon-Cobos, Konstantinos Vazaios, Piotr Waranecki, Marliek van Hoesel, Annelisa Cornel, Benjamin Schwalm, Norman Mack, Ella de Boed, Jasper van der Lugt, Stefan Nierkens, Marcel Kool, Eelco W. Hoving, Dennis S. Metselaar, Esther Hulleman

**Affiliations:** 1 Princess Máxima Center for pediatric oncology, Utrecht, Netherlands.; 2 Hopp Children’s Cancer Center Heidelberg (KiTZ), Heidelberg, Germany.; 3 Division of Pediatric Neurooncology, German Cancer Research Center (DKFZ) and German Consortium (DKTK), Heidelberg, Germany.; 4 University Medical Center Utrecht, Utrecht, Netherlands.; 5 Amsterdam UMC, Vrije Universiteit Amsterdam, Amsterdam, Netherlands.

**Keywords:** Immunology, Neuroscience, Oncology, Brain cancer, Cancer immunotherapy

## Abstract

Aggressive pediatric brain tumor models of ependymoma are highly sensitive to GD2-directed (CAR T) cell therapy

**To the Editor:** Ependymomas (EPNs) are a heterogeneous group of tumors that can arise across the entire CNS (1). At least 10 distinct molecular groups exist, with the ZFTA-fusion–positive supratentorial EPN (ST-ZFTA) and the posterior fossa group A (PF-A) being the most lethal and common in children. Notorious for their relapsing behavior and unresponsiveness to chemotherapy, EPNs pose a major challenge to the pediatric neuro-oncology field (2).

The glycoprotein disialoganglioside 2 (GD2) is considered an immunotherapeutic target in several solid tumors. For example, the GD2-targeting monoclonal antibody dinutuximab has shown promising results for high-risk pediatric neuroblastoma (3). Yet, in most CNS tumors the blood-brain-barrier prevents antibody entry, and thus requires a different therapeutic approach. In diffuse midline glioma (DMG), another aggressive pediatric brain malignancy, GD2-directed chimeric antigen receptor T cell (CAR T cell) therapy recently showed promising clinical results, with 1 patient even achieving complete remission (4). Here, we show that high-risk ST-ZFTA and PF-A EPN patient-derived models expressed GD2 and were highly sensitive to GD2–CAR T cell therapy, even exceeding the levels seen in DMG.

We established a series of patient-derived models of pediatric PF-A and ST-ZFTA EPN (Supplemental Figure 1A and Supplemental Figure 2A; supplemental material available online with this article; https://doi.org/10.1172/JCI193332DS1) following a previously established protocol (5). These models were validated by DNA methylation profiling and presented resistance to chemotherapeutics in a cytotoxicity experiment (Supplemental Figure 2, B and C). PMC-PFA-01 was obtained from a rare subtype of PF-A EPN, known as PFA-1f, which contains the H3K27M mutation, typically found in DMG (Supplemental Figure 1A). However, in these EPN cultures, we observed no correlation between H3K27M status and GD2 expression (6).

We assessed GD2 expression in our EPN models using an optimized cytospin immunofluorescence protocol. All 7 EPN models highly expressed GD2 (Figure 1A), in contrast to the GD2 negative control (an atypical teratoid/rhabdoid [ATRT] tumor model) (Figure 1, A and B). GD2 expression was also found on the original biopsy tissue from which models PMC-ZFTA-02 and PMC-PFA-03 were generated, and across multiple EPN tissues (Supplemental Figure 1, B and C).

We then quantified GD2 expression through flow cytometry in all EPN models, alongside 2 positive DMG controls (HSJD-DIPG-07 and VUMC-DIPG-G) and 2 negative controls (VUMC-ATRT-03 and VUMC-DIPG-10; ref. 6). All EPN cultures showed uniform, high GD2 membrane expression, comparable to or higher than in the DMG models (Figure 1B).

To test whether GD2 expression on EPN cells results in GD2-directed CAR T sensitivity, we exposed these models to allogeneic human GD2–targeting 4-1BB.CD3ζ CAR T cells, containing a 14G2α-binding domain, similar to CAR T cells currently in clinical trials (Figure 1C) (4). We observed high sensitivity and confirmed dose-dependent tumor death across all EPN models, exceeding the killing levels observed in the DMG positive controls (Figure 1D and Supplemental Figure 3A). Additionally, we monitored GD2–CAR T–mediated killing in real time, which demonstrated a rapid antitumor effect (Supplemental Figure 4 and the Supplemental Video found in the Mendeley Data Repository; https://data.mendeley.com/datasets/z8rrkwckxm/1).

As a marker for immune activation of the GD2–CAR T cells, IFN-γ was detected in a dose-dependent manner in the matched coculture supernatants, as quantified by ELISA (Figure 1E). GD2–CAR T cells did not produce IFN-γ or induce killing when presented with GD2 negative ATRT cells (Figure 1, D and E). Matched donor untransduced T cells also did not secrete IFN-γ, nor did they induce significant cell death (Figure 1, D and E), showing that both the tumor killing and IFN-γ secretion were GD2 dependent.

Finally, we evaluated the efficacy of GD2–CAR T cells in pilot in vivo experiments using the PF-A model DKFZ-BT232. Tumor progression was monitored by bioluminescence imaging (BLI) in cohorts with either medium or high tumor burden at treatment initiation (Figure 1F). In the medium tumor burden group, CAR T cell treatment induced a sustained suppression of tumor growth following a single intracerebroventricular (i.c.v.) injection after i.v. priming (Figure 1G). Additionally, GD2–CAR T cells conferred a survival advantage in the high-burden group (Figure 1H).

Overall, we demonstrate the potent antitumor efficacy of GD2–CAR T cells across multiple patient-derived high-risk EPN models, both in vitro and in vivo. These findings suggest a promising potential immunotherapeutic approach for pediatric EPN meriting further study.

## Funding support

- Horizon Europe/Marie Skłodowska-Curie Actions (co-fund project number 101081481).
- STOPbraintumors.org foundation
- (https://stophersentumoren.nl/).
- KiTZ Máxima Collaborative Grant, call 22.

## Supplementary Material

Supplemental data

Supporting data values

## Figures and Tables

**Figure 1:**
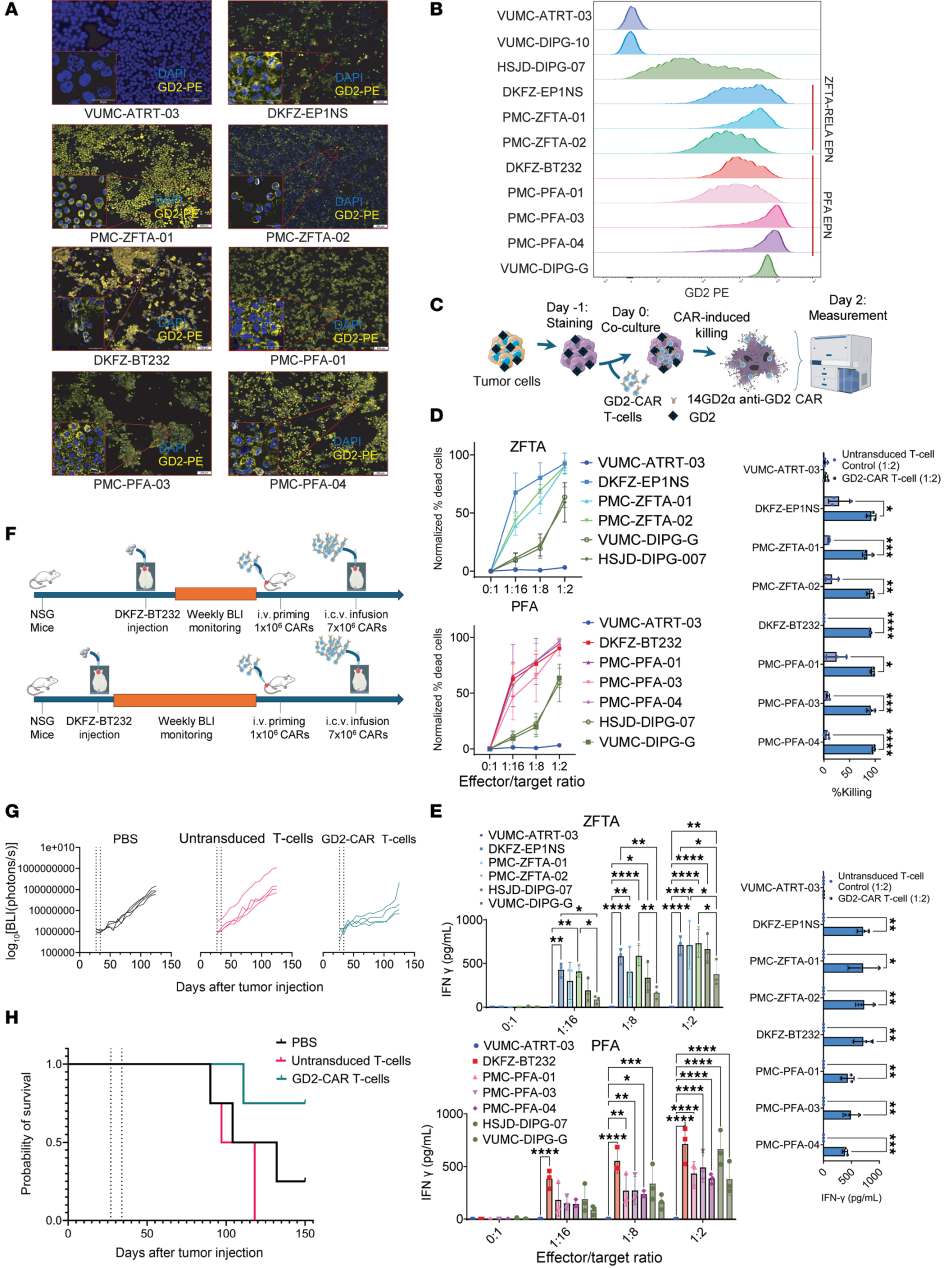
High-risk EPN models present the immunotherapeutic target GD2 and are sensitive to GD2–CAR T cell therapy in vitro and in vivo. (**A**) Immunofluorescence images of GD2 expression across 7 high-risk EPN models versus a GD2 negative control (VUMC-ATRT-03). Highlighted boxes indicate a higher objective (×63) of the indicated area (×20). Scale bars: 100 μm for ×20 objective and 25 μm for ×63 objective, respectively. (**B**) Representative flow cytometry histogram of GD2 membrane expression across all EPN models, 2 DMG positive controls (HSJD-DIPG-07 and VUMC-DIPG-G) and 2 negative controls (VUMC-DIPG-10 and VUMC-ATRT-03). The experiment was performed 3 times. (**C**) Schematic for the EPN-GD2–CAR T cell coculture. (**D**) Left: Killing curves showing GD2–CAR T cells mediating potent dose-dependent lysis in ST-ZFTA-RELA EPN (upper) and PF-A EPN (lower). Right: GD2–CAR T cells show significantly higher tumor killing than untransduced matched donor T cell controls. Each dot represents the average of 3 technical replicates over 3 independent repetitions. (**E**) Left: GD2–CAR T cells mediate potent IFN-γ secretion when presented with ST-ZFTA-RELA-EPN and PF-A EPN models, but not with a GD2 negative control (VUMC-ATRT-03). Right: GD2–CAR T cells show significantly higher IFN-γ secretion compared with untransduced matched donor T cell controls. Each dot represents the average of 3 technical replicates over 3 independent repetitions of the experiment. (**F**) Schematic for the in vivo experiments. (**G**) Tumor BLI signal over time of the medium tumor burden. (**H**) Kaplan-Meier survival curves of the mice with the starting higher tumor burden. In all experiments, *P <.05, **P <.01, ***P <.005, and ****P <.001, by multiple unpaired, 2-tailed Student’s *t* test (**D** and **E**, right) and 2-way ANOVA (**E**, left). Data represent the mean ± SD.

## Version 1 11/25/2025

In-Press Preview

## Version 2 01/16/2026

Electronic publication
