# Supplementary material for: CAR T cells targeting the glycoprotein GD2 show potent antitumor efficacy in high-risk ependymoma models
Source: J Clin Invest. 2025 Nov 25;136(2):e193332. doi: 10.1172/JCI193332 (PMC12807483; doi:10.1172/JCI193332)
Supplement: Supplemental data [file jci-136-193332-s002.pdf]

SUPPLEMENTARY MATERIAL AND METHODS

Sex as a biological variable

All information regarding the sex origin of the patient material can be found in the table on Supplementary Figure 1A. In regards of GD2 expression and CAR T-therapy response, all patient-derived models, regardless of sex, responded positively and sensitively to the therapy. Sex did also not play a role in the selection of any tissues and models. For the in vivo experiment, all mice were female, due to housing regulations. Additionally, the model engraftment was established in female mice originally.

Patient clinical history

Information regarding clinical history and molecular characteristics from the patient tissue of origin at the Máxima can be found in the table in Supplementary Figure 1A.

DNA-methylation clustering analysis and data availability

DNA methylation profiles were updated to the Swiss EPDiP Database (https://epidip.usb.ch), search numbers can be found atop their respective CNV plots, the lookup numbers are:

DKFZ-EP1NS: 208813900021_R08C01

PMC-ZFTA-01: 208813900022_R06C01

PMC-ZFTA-02: 208813900021_R07C01

DKFZ-BT232: 208813900022_R03C0

PMC-PFA-01: 208813900022_R01C01

PMC-PFA-03: 208813900021_R06C01

PMC-PFA-04: 208813900022_R04C01

Tissue processing and cell culturing

Fresh tissue biopsies and CUSA material were disaggregated through repeated mincing and were processed according to a brain tumor dissociation protocol (130-095-942, Miltenyi), and then cultured at 37°C and 5% CO_2 in modified Tumor Stem Medium (TSM), consisting of 48% DMEM/F12, 48% Neurobasal-A medium, 1% HEPES 1M, 1% MEM Non-essential amino acids, 1% 100 mmol/L sodium pyruvate, 1% GlutaMAX, supplemented with 2% B27, 1% N2 (all from Thermo Fisher Scientific), 20 ng/mL human EGF, 20 ng/mL human bFGF, 10 ng/mL human PDGF-AA, 10 ng/mL human PDGF-BB (all from Peprotech), and 5 IU/mL heparin (Leo Pharma, Ballerup, Denmark). To achieve long-term culture, adherent monolayers of the models were cultured in TSM with flasks and plates previously coated with poly-L-ornithine (Sigma-Aldrich P4957) and laminin (Sigma-Aldrich L2020), all according to an already established protocol (6). DKFZ-EP1NS and DKFZ-BT232 cells were a kind gift from Prof. Dr. Marcel Kool.

Of note, PMC-ZFTA-01 and PMC-PFA-04 showed no diagnostic match after methylation profiling. However, deviation from the original classifier diagnosis is a well-known side effect of culture establishment, since the methylation classifier was trained using tissues which are inherently heterogeneous (5). Nonetheless, the PMC-ZFTA-01 CNV methylation profile, as well as the short tandem repeat (STR) profile matched the original patient material, which was confirmed to be a ST-ZFTA EPN.

Immunofluorescence

To perform the cytospin protocol, tumor cells were made single cell suspension using accutase, and then counted with trypan blue (Thermo Fischer T10282,). Cells were then resuspended in cold PBS 2% FCS for a final concentration of $5*10^5$ cells/mL. 100 µL of ice-cold PBS were added to an already mounted cytospin cuvette and holder, and then centrifuged at 161 RCF for three

min to properly pre-wet the carton filter. Then, 200 μL of cell suspension were added into their respective cytospin cuvette and cells were spun at 161 RCF for seven min. Slides were air dried overnight. The following day, slides were washed three times with TBS 0.1% Tween 20 (Sigma-Aldrich 11332465001), and blocked using western blot (WB) blocking buffer for immunofluorescence (Rockland, MB-070) for 20 min. Subsequently, slides were stained with a pre-conjugated GD2-PE Antibody (BD Biosciences 562100) diluted 1:200 times in WB blocking buffer, and left overnight at 4°C in the dark. Finally, slides were washed three times with TBS 0.1% Tween 20 and three more times with PBS and stained for DAPI using NucBlue Staining Kit (Thermo Fischer R37606), according to the manufacturer's instructions. Slides were then Imaged in a DMI Thunder Microscope (Leica Microsystems) at 20x and 63x objectives accordingly.

In order to perform the GD2 staining on primary patient material, resection-derived FFPE materials from EPN patients were processed and stained for GD2 by the Prinses Maxima Center's diagnostic department, according to an already established protocol by Kailangiri et al. (PMID: 22374462, PMID: 39046881). The primary GD2 antibody was obtained from BD Pharmingen (Cat# 554272) and the secondary antibody was an anti-mouse Opal 520 reagent (Cat#FP1487001KT). Slides were then imaged in the research department in a DMI Thunder Microscope (Leica Microsystems) at 20x objective.

Flow cytometry

GD2 membrane stainings were obtained by making a single cell suspension of the tumor cells using accutase. Cells were then allotted to 96-well plates (~$1*10^5$ cells/well). Zombie Violet (BioLegend 423113) was used to exclude dead cells, and staining for GD2 was done using a pre-conjugated GD2 PE Antibody (BD Biosciences 562100), diluted 1:200 times in cold PBS. Cells

were then washed two times with ice-cold PBS and Analyzed on a Cytoflex LS (Supplementary Figure 3).

To perform the GD2-CAR T-cell co-culture, tumor cells were made single cell suspension using accutase at RT, counted with trypan blue and stained with 5 µM CellTrace Violet Cell Proliferation Kit™ (Thermo Fischer C34557). Cells were then plated in an already-coated Poly-L-Ornithine/laminin 96 well u-bottom Sarstedt plate, according to an already published protocol (5), at a density of $2*10^4$ cells/well. One day later, GD2-CAR T-cells and the matched donor untransduced T-cell controls were counted and added according to their respective effector to target (E:T) ratios. Finally, after 48 h, cells were centrifuged and supernatants were stored at -80°C for further analysis (ELISA), and tumor killing was measured using a 7AAD-Staining solution (Miltenyi 130-111-568) according to the manufacturer's instructions.

ELISA

ELISA was performed using an IFN-γ Human uncoated ELISA kit (Invitrogen 15531107) according to the manufacturer's instructions.

Incucyte live imaging

Tumor cells were made single cell using accutase at RT and counted with trypan blue. Cells were then plated in an already-coated Poly-L-Ornithine/laminin 96 well u-bottom Sarstedt plate, according to an already published protocol (5), at a density of $2*10^4$ cells/well. One day later, GD2-CAR T-cells and matched donor untransduced T-cells were counted and added at an E:T of 1:2. Killing was then monitored for 5 days using the IncuCyte® S3 Live-Cell Analysis System cell imaging system by Sartorius. GUI version 2023Arev2.

Statistics

All statistical analyses were performed using GraphPad Prism version 10.4.1 for Windows, (GraphPad Software, Boston, MA, USA, www.graphpad.com). Two-tailed unpaired Student's t-test was used to compare data from the untransduced controls vs the GD2-CAR T-cell at the highest ratio. Two-way-ANOVA was used to perform analysis in tumor-killing and IFN-γ secretion of the tumor models against a GD2-negative control (VUMC-ATRT-03). A p value less than 0.05 was considered significant. Non-linear regression fit was used to assess the anti-tumor effect of the therapeutic agents. Multilevel regression analysis (MLR) was performed to assess the survival benefit and control tumor growth in the *in vivo* experiments.

Study approval

Patient materials were collected at the Princess Máxima Center for Pediatric Oncology, according to national and institutional guidelines and in accordance with the declaration of Helsinki. All included patients provided written informed consent for participation in the biobank (International Clinical Trials Registry Platform, NL7744). Prinses Maxima Centrum voor kinderoncologie, Heidelberglaan 25, 3584 CS, Utrecht. All animal experiments were carried out according to governmental and institutional guidelines and approved by the Regierung-spräsidium Karlsruhe (permit number: 35-918.81/G-4/20). Department 35, Schlossplatz 4-6, 7631 Karlsruhe.

In vivo experiments

Overall, two different pilot experiments were performed. For the first trial, $5*10^5$ luciferase-expressing DKFZ-BT232 cells (kindly gifted by Prof. Dr. Marcel Kool) were orthotopically transplanted into the cerebellum of 12 NOD.Cg-*Prkdc*^{scid} Il2rg^{tm1Wjl}/SzJ mice purchased from

Charles River Laboratories (Sulzfed, Germany). Two weeks after injection, BLI was monitored weekly using the IVIS Lumina (Perkin Elmer) imaging system. Once the average BLI signal exceeded $1*10^7$ (large tumor burden), treatment was started. For the second trial, another 12 NOD.Cg-Prkdcscid Il2rgtm1Wjl/SzJ mice were transplanted identically. However, once the average BLI signal in this study reached $1*10^6$ (small tumor burden), treatment was started. All mice were then intravenously primed with $1*10^6$ GD2-CAR T-cells per mice, followed by an intraventricular injection of $7*10^6$ GD2-CAR T-cells per mice one week later. BLI was then monitored weekly. Both experiments were discontinued 150 days after tumor injection due to local legislation. During both intervention trials, no treatment related toxicities were observed.

Schematics

For the GD2-CAR T-cell co-culture, the symbols used were: cancerous-cell-5 icon by Servier https://smart.servier.com/ is licensed under CC-BY 3.0 Unported https://creativecommons.org/licenses/by/3.0/, T Cell. NIAID NIH BIOART Source. bioart.niaid.nih.gov/bioart/508, simple_receptor_2 icon by Helicase 11 undefined is licensed under CC-BY 4.0 Unported https://creativecommons.org/licenses/by/4.0/, NIAID Visual & Medical Arts. (07/10/2024). Flow Cytometer. NIAID NIH BIOART Source. bioart.niaid.nih.gov/bioart/160.

For the in vivo experiment set up, the symbols used were: T_Maze_Test_Mouse_Placed_in_the_Start_Arm icon by DBCLS https://togotv.dbcls.jp/en/pics.html is licensed under CC-BY 4.0 Unported https://creativecommons.org/licenses/by/4.0/, NIAID Visual & Medical Arts. (07/10/2024). TCell Receptor. NIAID NIH BIOART Source. bioart.niaid.nih.gov/bioart/510, NIAID Visual & Medical Arts. (07/10/2024). Lab Mouse. NIAID NIH BIOART Source. bioart.niaid.nih.gov/bioart/279, NIAID Visual & Medical Arts. (07/10/2024). T Cell. NIAID NIH BIOART Source. bioart.niaid.nih.gov/bioart/508

Acknowledgements

We thank the patients, parents and families for their kind and generous donations. We thank Dr. Lot Sewing for her clinical expertise and support. This work has been supported by, HE/MSCA co-fund project number 101081481, together with funding from the STOPbraintumors.org foundation (https://stophersentumoren.nl/), as well as the KiTZ Máxima Collaborative Grant, call 22.

Conflict of interest

The authors declare no conflict of interest.

A

Celline	Patient characteristics										Histology	Molecular characteristics								IHC					
	Patients age	M/F	Location of tumor	Primary tumor or replase	Location of relapse	Therapy previous to resection	Relapse In RT field	Total resection	Outcome	Localised / metastasized disease at diagnosis	Grade I, II or III	Methylation	Score	1q gain	6q loss	Mutations	Fusions	Losses	Amplifications	EZHIp	H3K27me3	H3K27M	Ki67	p65	L1CAM
PMC-PFA-01	5	F	posterior fossa	primary tumor	n.a.	dexamethason	n.a.	yes	in follow up	localised	Grade III	PFA,group 1A, subclass 1f	0.99	no	no	H3C3(NM 003531.2):c.83A>T (p.Lys28Met) and ASXL1.	none	none	none	n.a.	loss	positive	13%	n.a.	n.a.
PMC-PFA-03	4	F	frontal lobe	relapse	distant	RT	No	No	in follow up	metastasized	Grade III	PFA	0.98	yes	yes	none	none	none	1q	Strongly positive	loss	negative	60%	n.a.	n.a.
PMC-PFA-04	3	M	posterior fossa	primary tumor	n.a.	dexamethason	n.a.	yes	in follow up	localised	Grade II III	PFA	0.99	yes	no	none	none	none	1q	Strongly positive	loss	negative	5-10%	n.a.	n.a.
PMC-ZFTA-01	15	M	right frontal lobe	relapse	local	radiotherapy	yes	yes	in follow up	localised	Grade III	ZFTA:RELA fusion positive subtype A	0,99/0,88	no	no	none	C11orf95(NM_001144936.) exon 3 and RELA(NM_021975.4) exon 2	Homozygos loss of CDKN2A/B	none	n.a.	n.a.	n.a.	50%	positive	positive
PMC-ZFTA-02	6	M	left parietal lobe	primary tumor	n.a.	dexamethason	n.a.	yes	in follow up	localised	Anaplastic ependymoma grade III	ZFTA:RELA fusion-positive, subtype: A	0.99	no	no	none	C11orf95(NM_001144936.) exon 2 and RELA(NM_021975.4) exon 2	Homozygos loss of CDKN2A/B , partial loss 9p, loss chr 3,5,14q, 18, 22q	none	n.a.	n.a.	n.a.	30%	partially positive	strongly positive

B

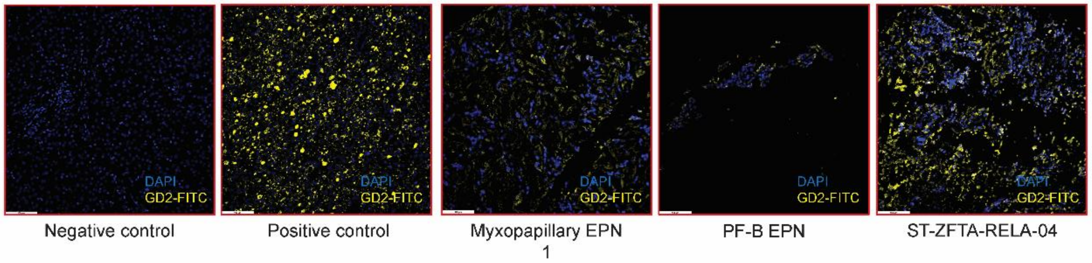

C

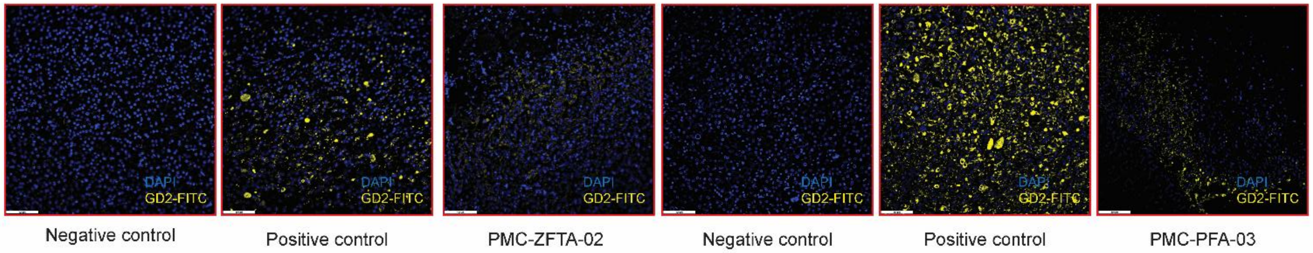

Supplementary Figure 1. *Patient characteristics and GD2 stainings across multiple EPN tissues.* ***A*** *Table containing each patient characteristics.* ***B*** *GD2 Patient stainings of an ependymoma tissue microarray.* ***C*** *GD2 Staining of original biopsy material from PMC-ZFTA-02 and PMC-PFA-03. Bar represents 100 µm. Each slide had its own in-slide positive and negative control. Positive controls are neuroblastoma tumor tissue. Negative controls are healthy liver tissue. An objective of 20x was used.*

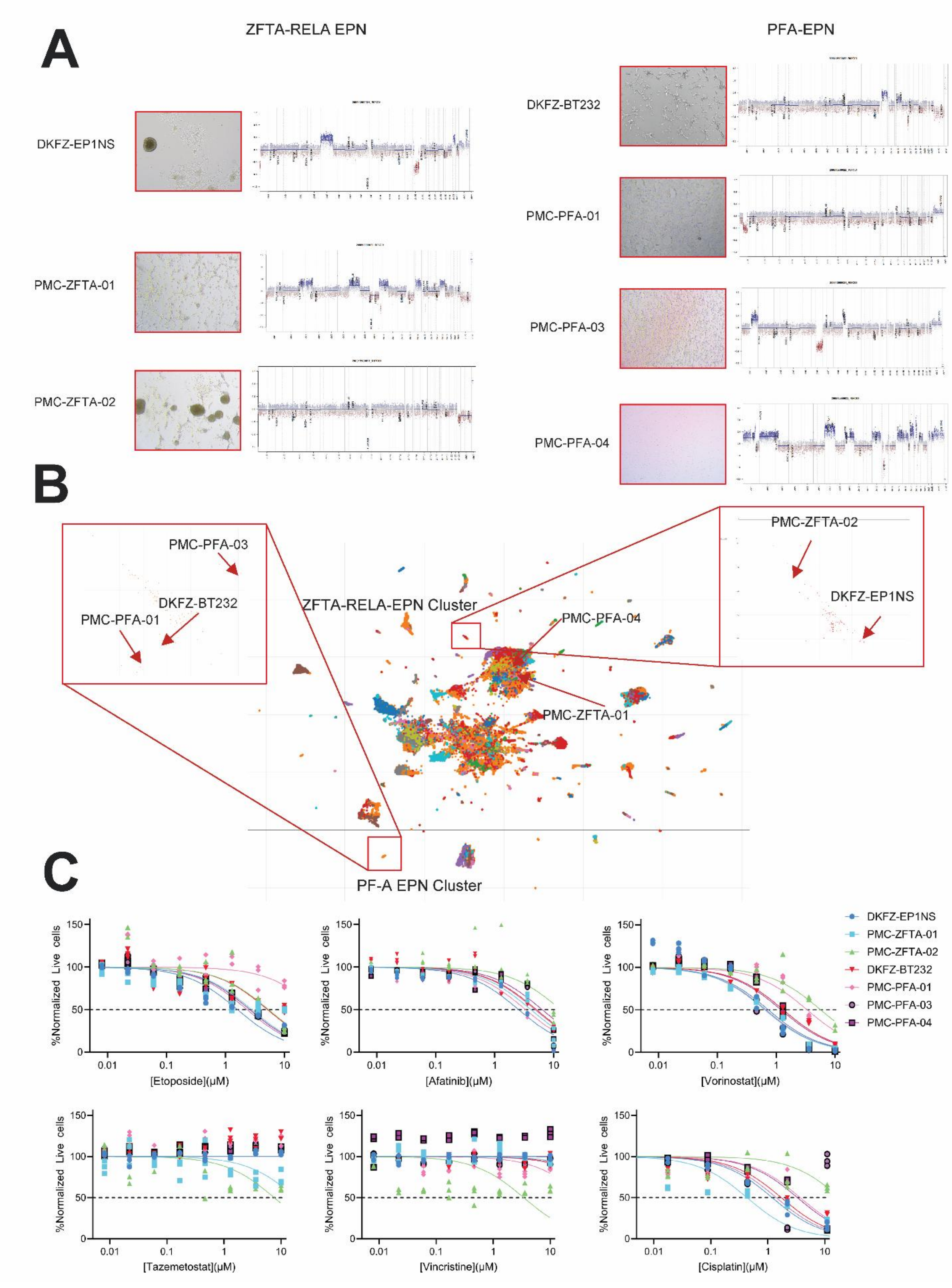
A
ZFTA-RELA EPN
PFA-EPN
DKFZ-EP1NS
PMC-ZFTA-01
PMC-ZFTA-02
DKFZ-BT232
PMC-PFA-01
PMC-PFA-03
PMC-PFA-04
B
ZFTA-RELA-EPN Cluster
PF-A EPN Cluster
PMC-PFA-03
DKFZ-BT232
PMC-PFA-01
PMC-ZFTA-02
DKFZ-EP1NS
PMC-PFA-04
PMC-ZFTA-01
C
%Normalized Live cells
[Etoposide](μM)
[Afatinib](μM)
[Vorinostat](μM)
[Tazemetostat](μM)
[Vincristine](μM)
[Cisplatin](μM)
DKFZ-EP1NS
PMC-ZFTA-01
PMC-ZFTA-02
DKFZ-BT232
PMC-PFA-01
PMC-PFA-03
PMC-PFA-04

1

Supplementary Figure 2. *Validation of the EPN models through DNA methylation and drug screening****. A*** *Pictures of EPN models along with their respective CNV plots.* ***B*** *DNA-methylation unbiased clustering analysis in the EPDiP Swiss database for tumor classification. PMC-ZFTA-01 shows up as PXA (0.55 score) due to location of the recurrence, short tandem repeats as well as CNV plot match those of the original patient material.* ***C*** *Measurement of the cell viability CellTiterGlo assay after treatment with increasing concentrations of chemotherapeutics etoposide, afatinib, vorinostat, tazemetostat, vincristine and cisplatin. Each dots represents a technical replicate.*

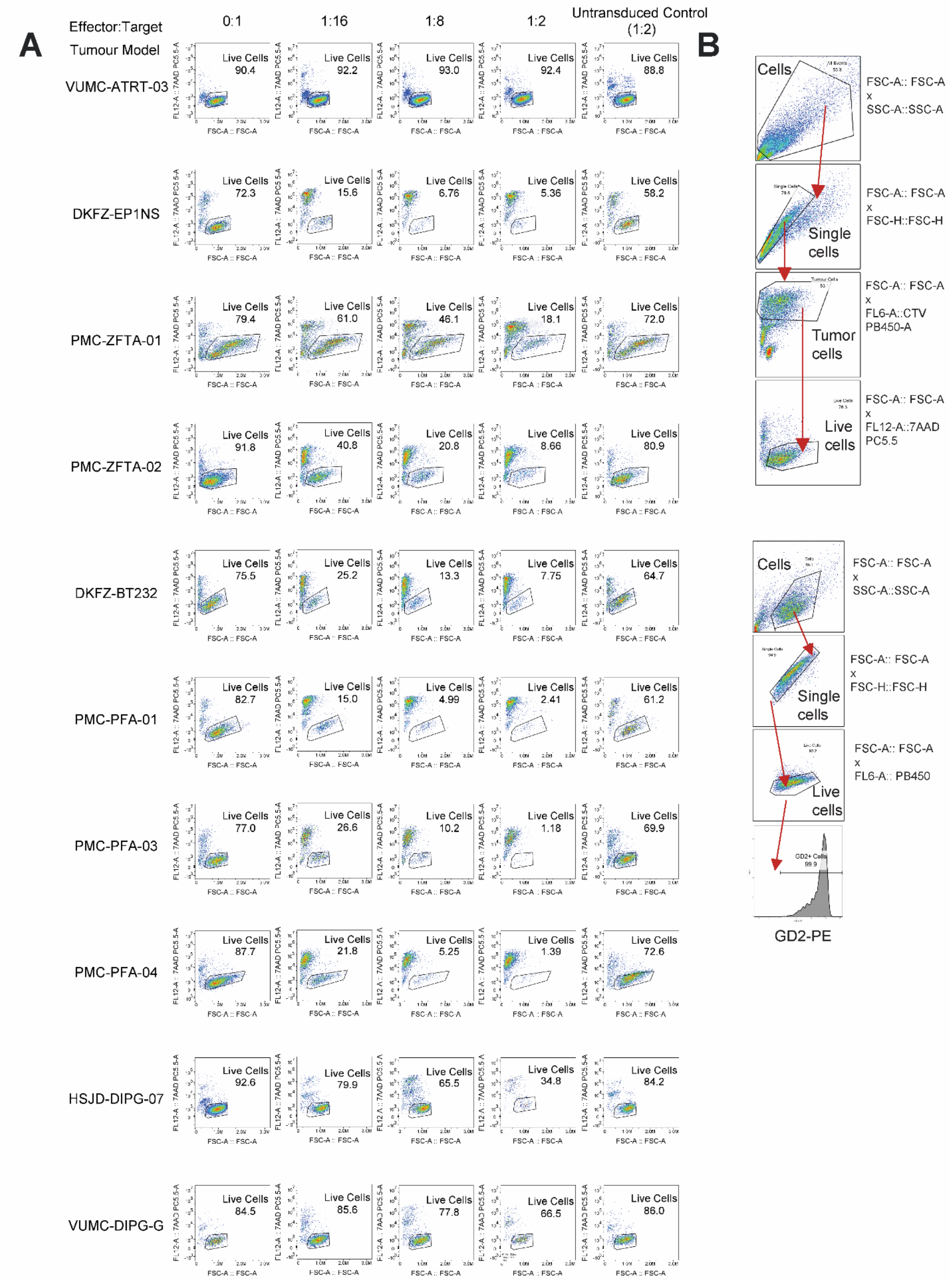
A
Effector:Target
Tumour Model
0:1
1:16
1:8
1:2
Untransduced Control (1:2)
VUMC-ATRT-03
Live Cells 90.4
Live Cells 92.2
Live Cells 93.0
Live Cells 92.4
Live Cells 88.8
DKFZ-EP1NS
Live Cells 72.3
Live Cells 15.6
Live Cells 6.76
Live Cells 5.36
Live Cells 58.2
PMC-ZFTA-01
Live Cells 79.4
Live Cells 61.0
Live Cells 46.1
Live Cells 18.1
Live Cells 72.0
PMC-ZFTA-02
Live Cells 91.8
Live Cells 40.8
Live Cells 20.8
Live Cells 8.66
Live Cells 80.9
DKFZ-BT232
Live Cells 75.5
Live Cells 25.2
Live Cells 13.3
Live Cells 7.75
Live Cells 64.7
PMC-PFA-01
Live Cells 82.7
Live Cells 15.0
Live Cells 4.99
Live Cells 2.41
Live Cells 61.2
PMC-PFA-03
Live Cells 77.0
Live Cells 26.6
Live Cells 10.2
Live Cells 1.18
Live Cells 69.9
PMC-PFA-04
Live Cells 87.7
Live Cells 21.8
Live Cells 5.25
Live Cells 1.39
Live Cells 72.6
HSJD-DIPG-07
Live Cells 92.6
Live Cells 79.9
Live Cells 65.5
Live Cells 34.8
Live Cells 84.2
VUMC-DIPG-G
Live Cells 84.5
Live Cells 85.6
Live Cells 77.8
Live Cells 66.5
Live Cells 86.0
FL12-A :: 7AAD PC5.5-A
FSC-A :: FSC-A
B
Cells
FSC-A:: FSC-A x SSC-A::SSC-A
Single cells
FSC-A:: FSC-A x FSC-H::FSC-H
Tumor cells
FSC-A:: FSC-A x FL6-A::CTV PB450-A
Live cells
FSC-A:: FSC-A x FL12-A::7AAD PC5.5
Cells
FSC-A:: FSC-A x SSC-A::SSC-A
Single cells
FSC-A:: FSC-A x FSC-H::FSC-H
Live cells
FSC-A:: FSC-A x FL6-A:: PB450
GD2+ Cells 99.9
GD2-PE

Supplementary Figure 3*. Representative raw dot plots of GD2-CAR T- cell co-culture and gating strategy.* ***A****: Representative raw dot plots for the quantification of killing.* ***B****: Gating strategy for GD2-CAR T-cell co-culture (upper) and of GD2 assessment (lower).*

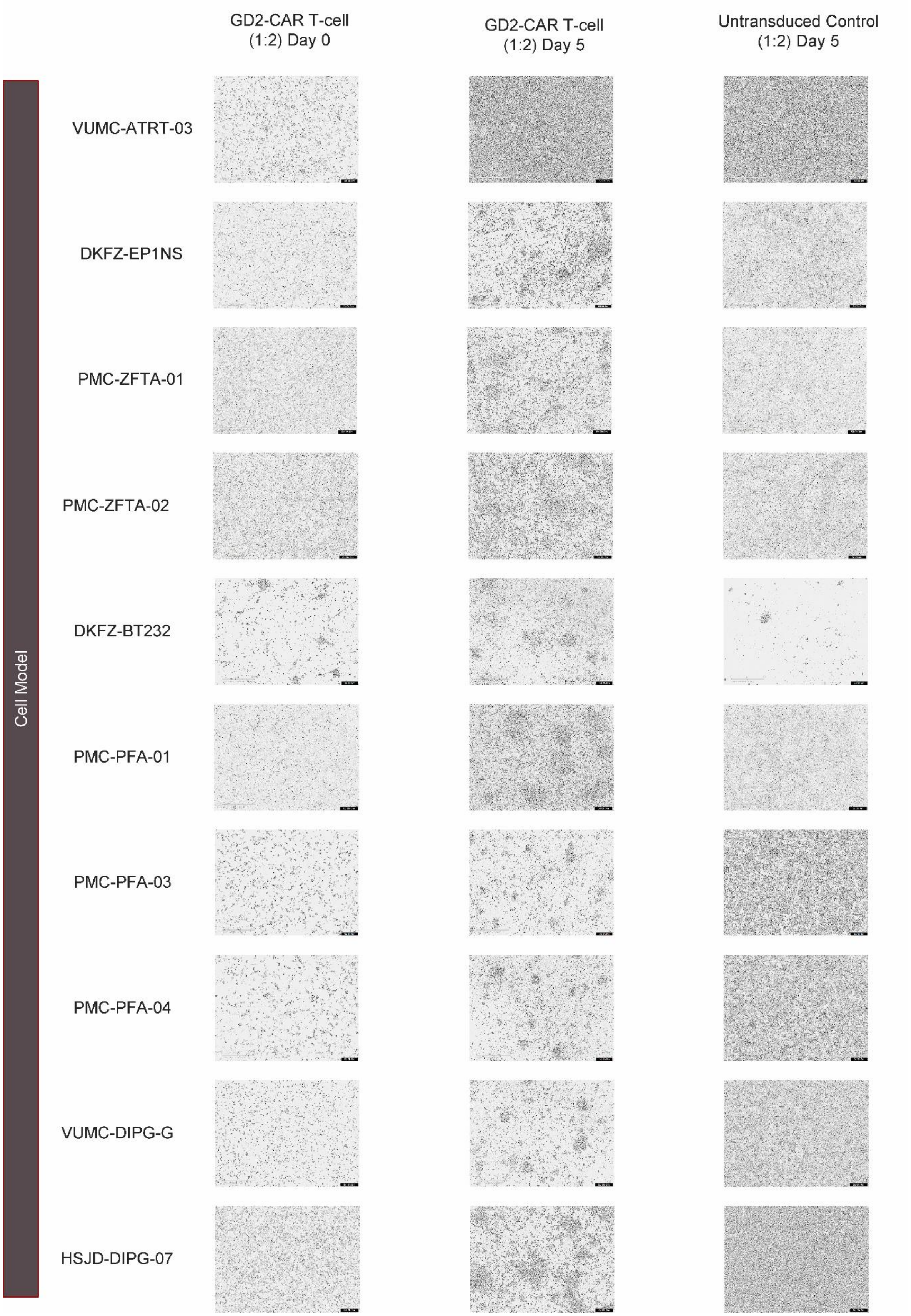
GD2-CAR T-cell (1:2) Day 0
GD2-CAR T-cell (1:2) Day 5
Untransduced Control (1:2) Day 5
Cell Model
VUMC-ATRT-03
DKFZ-EP1NS
PMC-ZFTA-01
PMC-ZFTA-02
DKFZ-BT232
PMC-PFA-01
PMC-PFA-03
PMC-PFA-04
VUMC-DIPG-G
HSJD-DIPG-07

1

Supplementary Figure 4*. Real-time tumor clearance of GD2-CAR T-cells. Image of all models in co-culture with either GD2-CAR T-cells (left and center) or matched donor untransduced T-cells (right). At day 0 and day 5 after co-culture. The videos showing the real-time tumor clearance can be found at Mendeley Repository URL: https://data.mendeley.com/datasets/z8rrkwckxm/1.*
